# Effects of microalgae as dietary supplement on palatability, digestibility, fecal metabolites, and microbiota in healthy dogs

**DOI:** 10.3389/fvets.2023.1245790

**Published:** 2023-09-27

**Authors:** Ana R. J. Cabrita, Joana Guilherme-Fernandes, Maria Spínola, Margarida R. G. Maia, Timur Yergaliyev, Amélia Camarinha-Silva, António J. M. Fonseca

**Affiliations:** ^1^REQUIMTE, LAQV, ICBAS, School of Medicine and Biomedical Sciences, University of Porto, Porto, Portugal; ^2^HoLMiR – Hohenheim Center for Livestock Microbiome Research, University of Hohenheim, Stuttgart, Germany; ^3^Institute of Animal Science, University of Hohenheim, Stuttgart, Germany

**Keywords:** digestibility, dog, fecal metabolites, microalgae, microbiota, palatability

## Abstract

The current trend of dog owners increasingly favoring the functional value of food to assure preventive health and wellbeing of their pets has been raising the interest in microalgae as natural additives with bioactive properties. However, scientific studies addressing the effects of microalgae supplementation in diets for dogs are scarce. This study aimed to evaluate the effects of dietary supplementation with three microalgae species (*Chlorella vulgaris*, *Nannochloropsis oceanica*, and *Tetradesmus obliquus*) on diet palatability, total tract digestibility, metabolizable energy content, fecal metabolites and microbiota of dogs. Twelve adult Beagle dogs were used in three two-bowl tests to compare the palatability of a commercial complete diet for adult dogs without (reference diet) and with 1.5% supplementation of each microalgae. From the results obtained, three digestibility trials were performed according to a replicated Latin square 3 × 3, with six adult Beagle dogs, three experimental periods of 10 days each, and three dietary supplementation levels of microalgae (0.5, 1.0, and 1.5%). In each trial, effects of microalgae supplementation levels on total tract digestibility, metabolizable energy content, fecal metabolites and microbiota of dogs were evaluated. First diet approached or tasted was not significantly affected by microalgae inclusion, but dogs showed a preference for the reference diet over the diets with 1.5% inclusion of *C. vulgaris* and *N. oceanica*, no difference being observed with 1.5% *T. obliquus*. In all digestibility trials, dietary supplementation with microalgae up to 1.5% did not greatly affected the dietary chemical composition and kept unaffected food intake, fecal output and metabolites, and digestibility of nutrients and energy. Compared with the reference diet, supplementation with *C. vulgaris* increased protein digestibility. Fecal characteristics and metabolites were affected by microalgae supplementation, being the effects dependent on the species. Fecal microbiota composition of dogs fed with microalgae-supplemented diets was modified by promoting the beneficial *Turicibacter* and *Peptococcus* genera associated with gut health and activation of the immune system. Overall, the results support *C. vulgaris*, *N. oceanica*, and *T. obliquus* as sustainable functional supplements that potentially enhance gastrointestinal health of dogs through the selective stimulation of microbiota without detrimental effects on food intake and digestibility.

## 1. Introduction

Microalgae are unicellular photosynthetic microorganisms rich in macro- and micronutrients and in bioactive compounds such as proteins, peptides, lipids, polyunsaturated fatty acids, pigments, minerals, and polysaccharides (1). Microalgae have been suggested to be a viable strategy for a more sustainable food sector due to their ability to convert inorganic and organic carbon sources into nutrient-rich biomass more efficiently than terrestrial plants and require less land and water resources (2). However, to take advantage of microalgae’s potential in pet food, their market availability at reasonable prices is crucial. The large-scale cultivation of some microalgae species developed in recent years have promoted their use in animal feeding, comprising 19% of the European production (3). In the pet food sector, the demand for microalgae is expected to continue growing (4), being microalgae currently used mainly as additives (generally with declared levels lower than 0.5%) in balanced food and in supplements or treats to benefit from their functional value as immunomodulatory (5), antioxidant, antimicrobial, and anti-inflammatory effects (6). The current tendency of dog owners increasingly choose the food that provides increased nutrition, health and wellness to their pets and the growing numbers of senior animals contributes to the raised interest in microalgae in the pet food market in recent years (7).

Despite the recently unveiled potential of microalgae as alternative and more sustainable food for dogs (8) and the commercial availability of microalgae-based pet food products, scientific studies addressing the effects of microalgae supplementation of diets for dogs are scarce. Positive effects of 0.4% *Schizochytrium* sp. dietary inclusion has been reported on palatability, protein digestibility and oxidative stability of diets, and phagocytic cell numbers of dogs (9), and to contribute to healthy brain function in a canine model of senescence (10). In dogs with the hematopoietic system damaged by irradiation, a polysaccharide of *Arthrospira platensis* (formerly *Spirulina platensis*) included at 0.08% (corresponding to 10.7% whole, dried spirulina) increased white blood cell number (11). A dietary inclusion of 0.2% spray-dried *A. platensis* has been shown to have an immune-stimulation effect as a higher vaccine response and higher levels of fecal IgA were observed in supplemented dogs compared to the control group (5). Although palatability of nutraceuticals can greatly impact convenience of administration and owner compliance (12), only scarce studies have assessed the palatability of microalgae supplemented diets.

Despite the known effects of some microalgae species on gut microbiota (13), to the best of our knowledge, there are no studies evaluating effects on dog gut microbiota, with the only exception of the work performed by Delsante et al. (14) using an *in vitro* canine gut model. In this study, microalgae species (*A. platensis*, *Haematococcus pluvialis*, *Phaeodactylum tricornutum*, and *Chlorella vulgaris*) have been shown to affect microbial saccharolytic activities and fecal bacterial composition, though in a less extent than anticipated from other species.

To deeper the knowledge on the potential of microalgae as supplements for dog feeding, this study aimed to evaluate the effects of different supplementation levels of *C. vulgaris*, *Nannochloropsis oceanica*, and *Tetradesmus obliquus*, among the top produced species in Europe (3), on palatability, apparent total tract digestibility of nutrients and energy (ATTD), metabolizable energy (ME) content, fecal metabolites and microbiota of dogs.

## 2. Materials and methods

Trials were approved by the Animal Ethics Committee of School of Medicine and Biomedical Sciences, University of Porto (Permit No. 344). Animal handling and procedures were performed in accordance with good animal welfare practices (European Union Directive 2010/63/EU) by trained scientists in laboratory animal science (FELASA, category C). All dogs were subjected to physical and clinical examinations to check their suitability to participate in the trial. Dogs were healthy throughout the length of the study, with no clinical signs of disease.

### 2.1. Animals and housing

Twelve healthy Beagle dogs (mean age: 2.2 ± 0.03 years; mean body weight (BW): 12.6 ± 1.55 kg), six males and six females, housed at the kennel of the School of Medicine and Biomedical Sciences, University of Porto, were used in the experimental protocols. Sample size was defined in accordance with the minimum number of animals recommended by the FEDIAF (15) for digestibility experiments. Animals were housed in pairs in environmentally enriched and communicating boxes with sliding doors to allow their individual feeding. Each box comprises an interior and an exterior area of 1.8 and 3.5 m^2^, respectively. Animals were leash walked once a day for at least 30 min and had free access to an outdoor park area between daily meals to exercise and socialize. During the feces collection period of the digestibility trials, animals were housed individually, having supervised access to an outdoor park between daily meals to ensure the collection of individual feces. Temperature and relative humidity of the kennel were monitored daily.

### 2.2. Palatability trials

A high economy commercial extruded complete diet for adult dogs (SilverDog, Sorgal Pet Food, Ovar, Portugal) widely available in retail stores as supermarkets and hypermarkets and including (label information) cereals, animal meals, wheat bran and beet pulp, and without the inclusion of microalgae was used as a reference diet. Three two-bowl tests (16) were conducted to determine palatability by the pairwise comparison of the reference diet with the reference diet supplemented with 1.5% of each microalgae species in substitution of the reference diet. The three studied commercially available microalgae species were produced locally in photobioreactors (Allmicroalgae – Natural Products, S.A.; Pataias, Portugal) and were provided as a spray dried powder in airtight bags protected from light. Microalgae were added to the reference diet immediately before offering the mixture to each dog, thus not being included in the reference diet kibble. After overnight fasting and for two consecutive days, the animals (*n* = 12) were given the choice between the two diets in two different bowls (45 cm apart). The position of the bowls was switched between days to control side bias. Daily feed allowance was calculated to supply the energy requirements of dogs (15). The bowl that was first approached and the diet that was first tasted were recorded. Trials ended after 30 min or until animals consumed all the food available in one bowl. Food offered and food residues were weighed to calculate the intake ratio of the two diets.

### 2.3. Digestibility trials

All digestibility trials were conducted using the method of total fecal collection. The *in vivo* digestibility of the reference diet was firstly determined using 12 animals for 10 days following the guidelines of FEDIAF (15). Then, three digestibility trials were conducted to evaluate the effects of increasing levels of dietary supplementation (0.5, 1.0, and 1.5% in substitution of the reference diet) of *C. vulgaris*, *N. oceanica*, and *T. obliquus*. The levels of microalgae supplementation were defined after evaluating the palatability of diets with 1.5% inclusion of each microalgae. Microalgae were added to the reference diet immediately before offering the mixture to each dog, thus not being included in the kibble.

The three trials were designed according to a replicated Latin square 3 × 3, with six animals (three males and three females, selected from the 12 animals used for the evaluation of the *in vivo* digestibility of the reference diet), three experimental periods, and three dietary inclusion levels (0.5, 1.0, and 1.5% in substitution of the reference diet). Each period lasted 10 days, with 5 days for diet adaptation, and 5 days for total feces collection. In all trials, daily food allowance was calculated to meet the ME requirements according to the ideal BW of individuals [ME (kcal/day) = 110 × BW^0.75^; (15)], and adjusted to body condition score assessed through a scale from 1 to 9 (17). Animals were individually fed twice a day the daily ration in two equal meals, at 8:30 h and 17:00 h, and had free access to fresh water at all times.

During total feces collection period, the number of defecations was recorded every day and individual fecal samples collected from the concrete floor were scored using a 5-point scale [from 1, corresponding to watery diarrhoea, to 5, corresponding to powdery hard mass pellets; (18)], weighed, mixed, subsampled at different locations and immeditaley frozen at −20°C until the end of the trials to perform analysis of chemical composition, fecal pH, ammonia-N and volatile fatty acids concentrations, and fecal microbiota. Analysis were carried out in feces composited by period and dog.

### 2.4. Analytical procedures

#### 2.4.1. Proximate analysis

The proximate composition of the reference diet (dried at 65°C and 1 mm milled), of the microalgae species and of the feces (dried at 65°C and 1 mm milled) was analyzed in duplicate according to official methods (19), as described by Cabrita et al. (8). Briefly, samples were analyzed for dry matter (DM; ID 934.01), ash (ID 942.05), total lipids, and Kjeldahl N (ID 990.03). Crude protein (CP) was calculated as Kjeldahl *N* × 6.25. Neutral detergent fiber (with α-amylase and without sodium sulfite, NDF) was analyzed in all samples and expressed exclusive of residual ash (20). Acid detergent fiber (ADF) of the reference diet and microalgae species were also analyzed and expressed exclusive of residual ash (21). For microalgae, hydrolyzed samples were filtered through a glass microfiber filter (Whatman GF/A, 1.6 μm porosity, Merck KGaA, Darmstadt, Germany). For the reference diet and microalgae, starch content was determined according to Salomonsson et al. (22) and gross energy (GE) using an adiabatic bomb calorimeter (Werke C2000, IKA, Staufen, Germany). The chemical composition of the reference and experimental diets supplemented with increasing levels of each microalga (0.5, 1.0, and 1.5%) in substitution of the reference diet is presented in Tables 1, 2; chemical composition of the studied microalgae species and a more detailed characterization of the reference and the experimental diets being presented in Supplementary Tables S1, S2.

**Table 1:** Proximate composition (g kg^−1^ dry matter, DM) and gross energy (MJ kg^−1^ DM) of the reference and experimental diets with inclusion of microalgae in substitution of the reference diet.

	Diet									
	Reference	*Chlorella vulgaris*			*Nannochloropsis oceanica*			*Tetradesmus obliquus*		
		0.5%	1.0%	1.5%	0.5%	1.0%	1.5%	0.5%	1.0%	1.5%
DM, g/kg	924	924	925	925	924	925	925	924	925	925
Ash	125	125	125	125	126	127	128	125	125	125
Crude protein	252	253	254	255	252	252	252	253	254	254
Total lipids	81.0	81.1	81.2	81.3	81.3	81.6	81.9	81.0	81.0	81.0
Neutral detergent fiber	228	228	227	227	228	227	227	228	228	228
Acid detergent fiber	56.7	56.9	57.1	57.3	56.6	56.6	56.5	57.0	57.3	57.6
Starch	311	310	308	307	309	308	306	309	308	306
Gross energy	18.3	18.3	18.3	18.3	18.3	18.3	18.3	18.3	18.3	18.3

**Table 2:** Essential amino acids, macro- and trace elements, and selected fatty acids content (g kg^−1^ dry matter, DM) of the reference and experimental diets with inclusion of microalgae in substitution of the reference diet.

	Diet									
	Reference	*Chlorella vulgaris*			*Nannochloropsis oceanica*			*Tetradesmus obliquus*		
		0.5%	1.0%	1.5%	0.5%	1.0%	1.5%	0.5%	1.0%	1.5%
Essential amino acids										
Arginine	20.5	20.6	20.8	20.9	20.5	20.5	20.5	20.5	20.6	20.6
Histidine	6.43	6.45	6.46	6.48	6.42	6.42	6.41	6.42	6.41	6.40
Lysine	16.0	16.2	16.4	16.6	16.0	16.1	16.1	16.1	16.1	16.2
Threonine	10.5	10.6	10.7	10.8	10.5	10.5	10.6	10. 6	10.6	10.7
Isoleucine	9.63	9.69	9.75	9.81	9.64	9.65	9.66	9.66	9.69	9.72
Leucine	21.2	21.3	21.4	21.5	21.2	21.2	21.2	21.2	21.3	21.3
Valine	16.2	16.3	16.3	16.4	16.2	16.2	16.2	16.2	16.2	16.3
Methionine	3.68	3.72	3.75	3.79	3.69	3.70	3.73	3.70	3.72	3.74
Methionine + cystine	8.24	8.26	8.29	8.31	8.23	8.22	8.21	8.24	8.25	8.25
Phenylalanine	12.2	12.3	12.4	12.5	12.2	12.2	12.2	12.3	12.3	12.3
Phenylalanine + tyrosine	19.0	19.2	19. 4	19.6	19.0	19.1	19.1	19.1	19.2	19.3
Fatty acids										
C16:0	20.0	20.0	20.0	19.9	20.0	20.0	20.0	20.0	20.0	19.9
C16:1 *n*-7	2.42	2.42	2.41	2.40	2.52	2.61	2.71	2.42	2.41	2.40
C18:0	8.16	8.13	8.10	8.07	8.12	8.09	8.05	8.12	8.09	8.05
C18:1 *n*-9	29.4	29.3	29.1	29.0	29.3	29.2	29.0	29.3	29.2	29.0
C18:2 *n*-6	22.0	22.0	21.9	21.8	21.9	21.8	21.7	21.9	21.8	21.7
C18:3 *n*-3	1.20	1.29	1.37	1.46	1.19	1.19	1.19	1.29	1.37	1.46
C20:4 *n*-6	0.024	0.024	0.024	0.024	0.024	0.024	0.024	0.023	0.023	0.023
C20:5 *n*-3 (EPA)	0.028	0.028	0.029	0.029	0.117	0.206	0.295	0.028	0.029	0.030
C22:6 *n*-3 (DHA)	0.122	0.123	0.123	0.123	0.122	0.122	0.121	0.123	0.123	0.124
Macro elements										
Na	3.13	3.12	3.10	3.09	3.30	3.48	3.65	3.12	3.12	3.11
K	6.03	6.04	6.05	6.07	6.09	6.16	6.22	6.06	6.10	6.13
Mg	0.430	0.435	0.435	0.436	0.451	0.469	0.486	0.435	0.435	0.436
Ca	15.5	15.4	15.3	15.3	15.4	15.3	15.2	15.4	15.3	15.3
P	21.0	21.0	21.0	21.1	21.0	20.9	20.9	21.0	21.0	21.0
Ca:P ratio	0.736	0.733	0.730	0.727	0.732	0.729	0.726	0.733	0.731	0.728
Trace elements (mg kg^−1^ DM)										
Fe	135	138	140	143	136	137	137	149	164	178
Mn	45.6	46.2	46.8	47.4	45.6	45.5	45.5	45.9	46.2	46.6
Cu	13.5	13.6	13.6	13.7	13.5	13.5	13.5	13.5	13.4	13.4
Zn	177	178	179	180	176	176	175	177	176	176
Se	0.350	0.349	0.348	0.347	0.355	0.360	0.365	0.350	0.350	0.349

#### 2.4.2. Amino acid analysis

Amino acid analysis were performed in duplicate. Samples of the reference diet and microalgae species were hydrolyzed with 6 M HCl solution at 116°C for 48 h and precolumn derivatized with Waters AccQ Fluor Reagent (6-aminoquinolyl-N-hydroxysuccinimidyl carbamate) according to the AccQ Tag method (Waters, Milford, MA). Analyses were carried out by ultra-high-performance liquid chromatography on a Waters reversed-phase amino acid analysis system with norvaline as internal standard. The resulting peaks were analysed with EMPOWER software [Waters; (23)].

#### 2.4.3. Fatty acid analysis

Fatty acids of the reference diet and microalgae samples were converted to fatty acid methyl esters by acid-catalyzed transesterification with methanolic HCl (24) and analyzed by gas chromatography as reported by Maia et al. (25). Nonadecanoic acid (Matreya LLC, Pleasant Gap, PA) was used as internal standard. Fatty acids were identified by comparing retention times to commercially available standards and quantified with the internal standard.

#### 2.4.4. Mineral analysis

Minerals and trace elements of microalgae and reference diet samples were determined in triplicate as described by Cabrita et al. (26). Briefly, reference diet and microalgae samples were mineralized (MLS 1200 Mega high-performance microwave digestion unit, Milestone, Sorisole, Italy) and sample solutions analyzed by inductively coupled plasma-mass spectrometry (ICP-MS; iCAP Q ICP-MS instrument, Thermo Fisher Scientific, Waltham, MA) and flame atomic absorption spectrometry (FAAS; AAnalyst 200 FAAS instrument, PerkinElmer, Shelton, CT). Calibration standards from 1,000 mg/L single-element standard stock solutions (Fluka, Buchs, Switzerland) were diluted with HNO_3_ 0.2% (v/v) for FAAS analysis. For ICP-MS determinations, internal standards and tuning solutions from diluted commercial solutions were prepared (Periodic table mix 3 for ICP-MS, TraceCERT^®^, Sigma-Aldrich, Buchs, Switzerland; custom solution, SCP Science, Baie D’Urfé, QC, Canada).

#### 2.4.5. Fecal pH, ammonia-N, and volatile fatty acids concentrations

Analysis was run in duplicate. Thawed feces were diluted to 1:10 (w/v) in 20 mL of water, sonicated, and incubated for 10 min at room temperature. The pH was determined using a potentiometer (pH and Ion-Meter GLP 22, Crison, Barcelona, Spain). The concentration of ammonia-N was determined using the method of Smith et al. (27) adapted to dog feces. Briefly, 1 g of feces were solubilized in 10 mL of KCl 2 M, centrifuged for 60 min at 5200 × g at 4°C, and the supernatant filtered using a 0.45 μm pore size polyethersulfone syringe filter (FILTER-LAB, Barcelona, Spain). Forty μL of supernatant were mixed with 40 μL of water, 2.5 mL of phenol solution and 2 mL of alkaline hypochlorite solution. After incubation for 10 min at 37°C and 40 min in the dark at 22°C, the absorbance of samples was read at 550 nm in a SynergyTM HT Multimode plate reader (BioTek^®^ Instruments Inc., Winooski, VT). An ammonia solution (32 mg/dL) was used as standard. For volatile fatty acids (VFA) analysis, feces were acidified with ortho-phosphoric acid solution, centrifuged for 60 min at 2360 × g at 4°C, and the supernatant analyzed by gas chromatography as described by Pereira et al. (28).

#### 2.4.6. Fecal microbiota

For microbiota analysis, fecal DNA in thawed samples was extracted by FastDNA^™^ Spin Kit for soil (MP Biomedicals, Irvine, CA) and used for 16S library preparation, targeting bacterial V1–V2 hypervariable regions (29). Unique barcodes (6-nt) were attached to forward primers, and index adapters were linked to reverse. Amplicons were obtained by two-step polymerase chain reaction (PCR). Briefly, 1 μL of DNA was added for the first PCR, in a 20 μL reaction with 0.2 μL of PrimeSTAR HS DNA polymerase (TaKaRa, Beijing, China) and 0.5 μL of each primer. The second PCR, which used 1 μL of the first PCR as a template, ran in a total volume of 50 μL. An initial denaturation at 95°C for 3 min was followed by 15 cycles (first PCR) or 20 cycles (second PCR) of denaturation at 98°C for 10 s, subsequent annealing at 55°C for 10 s, extension step at 72°C for 45 s and a final extension for 2 min at 72°C. Amplicon normalization was performed by the SequalPrep Normalization Kit (Invitrogen Inc., Carlsbad, CA) and sequenced with the 250 bp paired-end Illumina NovaSeq 6,000 platform.

Sequences were demultiplexed with Sabre^1^ and analyzed using Qiime2 (30). Primers were trimmed with q2-cutadapt plugin (31). Denoising and merging were accomplished by the q2-dada2 (32). Taxonomic classification of amplicon sequence variants (ASVs) was carried out with VSEARCH-based consensus (33) and pre-fitted sklearn-based classifiers (34) against the Silva database [v138.1, 16S 99%; (35)], for which reference reads and corresponding taxonomies were prepared by RESCRIPt (36). A phylogenetic tree was constructed by the q2-phylogeny, utilizing MAFFT [v7.3; (37)] and FastTree [v2.1; (38)]. For calculation of diversity metrics, the dataset was rarefied to 15,000 reads. Alpha diversity was assessed by Shannon’s entropy (39) and beta diversity by Bray–Curtis (40) distances. Beta diversity ordination was carried out by principal-coordinate analysis [PCoA; (41)]. Alpha diversity metrics were compared by Wilcoxon test (42), and beta diversity distances by the adonis test [999 permutations; (43)]. Differentially abundant genera (only for counts of genera with relative abundance ≥1% and prevalence ≥10%) were detected by ALDEx2 (44). All *p*-values obtained from multiple comparisons were adjusted using the Benjamini-Hochberg procedure (45).

Raw sequences are available at the European Nucleotide Archive (ENA) under accession number PRJEB61064.

### 2.5. Calculations and statistical analysis

Diet first-approach and first taste results were submitted to the Chi-square test and the intake ratio (intake of reference diet or diet with 1.5% microalgae inclusion / total intake of both diets) to the Student’s *t*-test, both at 5% probability level (*n* = 24).

Fecal production (%) was calculated as:


$$\mathrm{Fecal}\;\mathrm{production}\;\left(\%\right)=\frac{\mathrm{dried}\;\mathrm{feces}\;\mathrm{output}\;\left(\frac{g}{d}\right)}{\mathrm{dry}\;\mathrm{matter}\;\mathrm{intake}\;\left(\frac{g}{d}\right)}\times 100$$

Apparent total tract digestibility (%) of the reference diet and diets with microalgae inclusion was calculated using the equation as follows:


$$\mathrm{ATTD}\;\left(\%\right)=\frac{\mathrm{nutrient}\;\mathrm{intake}\;\left(\frac{g}{d}\right)-\mathrm{fecal}\;\mathrm{output}\;\left(\frac{g}{d}\right)}{\mathrm{nutrient}\;\mathrm{intake}\;\left(\frac{g}{d}\right)}\times 100$$

The following equation calculated ME content (MJ/kg DM) of diets (46):


$$\mathrm{ME}\;\left(\frac{MJ}{\mathrm{kg}\;DM}\right)=\frac{\begin{array}{l} \left(GE\;\mathrm{intake}\;\left(\frac{MJ}{d}\right)-\mathrm{fecal}\;GE\;\left(\frac{MJ}{d}\right)\right)-\\ \;\left(CP\;\mathrm{intake}\;\left(\frac{g}{d}\right)-CP\;\mathrm{excretion}\;\left(\frac{g}{d}\right)\right)\times 1.25 \end{array}}{DM\;\mathrm{intake}\;\left(\frac{g}{d}\right)}$$

For each digestibility trial, data on food and nutrient intake, fecal production and characteristics, ATTD, fecal pH, and ammonia-N and VFA concentrations were analyzed according to a replicated 3 × 3 Latin square. The model included the fixed effects of the square, dog within the square, period, level of microalgae inclusion and the residual error (SAS 2021, release 3.1.0., SAS Institute, Cary, NC, United States). When differences were significant (*p* < 0.05), the least significant difference test was used to compare means.

As experimental period had a minor effect on the parameters measured in the digestibility trials, and regarding ATTD, only ATTD of DM, organic matter and NDF were affected by period in the trial with *C. vulgaris* supplementation, a *t*-test was performed to compare the reference diet with diets with inclusion of microalgae (SAS 2021, release 3.1.0.) to mimic the at-home scenario of dog owners changing the diet offered to their animals, thus understand the perceived effects. For each microalgae under study, data from the six dogs collected during the digestibility trial on the reference diet were used for comparison.

## 3. Results

### 3.1. Palatability trials

The results of the two-bowl tests are shown in Table 3. First diet approached and first diet tasted were not affected by microalgae inclusion (*p* > 0.05). Dogs showed a preference for the reference diet in comparison with diets with 1.5% inclusion of *C. vulgaris* (*p* = 0.003) and *N. oceanica* (*p* < 0.001), no difference being observed for intake ratio with the inclusion of 1.5% of *T. obliquus* (*p* = 0.121).

**Table 3:** First approach and taste, and intake ratio of reference diet and experimental diets supplemented with 1.5% of microalgae in substitution of the reference diet.

	*Chlorella vulgaris*		*p*-value	*Nannochloropsis oceanica*		*p*-value	*Tetradesmus obliquus*		*p*-value
	0%	1.5%		0%	1.5%		0%	1.5%	
First approach	11	13	0.683	15	9	0.221	12	12	1.00
First taste	12	12	1.00	14	10	0.414	13	11	0.683
Intake ratio	0.618	0.382	0.003	0.830	0.170	<0.001	0.609	0.391	0.121

### 3.2. Chemical composition of reference and experimental diets

The chemical composition of the commercial complete diet for adult dogs used as the reference diet presented 252 g/kg CP, 81.0 g/kg total lipids, 16.0 g/kg Lys, 22.0 g/kg C18:2 *n*-6, and 0.736 Ca:P ratio. Dietary supplementation with microalgae up to 1.5% did not greatly affect the chemical composition of diets, with observed minor changes reflecting the chemical composition of microalgae species (Tables 1, 2).

### 3.3. Digestibility trials

All dogs remained healthy throughout the studies. No weight loss, vomiting, or diarrhea were observed. As food allowance was adjusted according to ideal BW and body condition score, BW of dogs remained unchanged during all the trials.

#### 3.3.1. Experiment 1. *Chlorella vulgaris*

Level of *C. vulgaris* supplementation did not affect food and nutrient intake and fecal output (*p* > 0.05), but number of defecations was higher when dogs were fed the diet supplemented with 1.0% of *C. vulgaris* (*p* = 0.026; Table 4). Digestibility of DM, nutrients and energy and ME content was not affected by *C. vulgaris* supplementation level, except ATTD of NDF that tended (*p* = 0.058; Table 4) to be higher with 1.5% supplementation. Fecal pH, and ammonia-N and VFA concentrations were not affected by *C. vulgaris* supplementation (*p* > 0.05; Table 4).

**Table 4:** Experiment 1. Effects of increasing supplementation levels of *Chlorella vulgaris* on food, gross energy and nutrient intake (dry matter, DM, basis), fecal output and characteristics, apparent total tract digestibility (ATTD, %), metabolizable energy content (MJ kg^−1^ DM), and fecal pH, concentration of ammonia-N (g kg^−1^ DM), and concentration of volatile fatty acids (μmol g^−1^ DM).

	Diet			SEM	*p*-value
	0.5%	1.0%	1.5%		
Food intake					
g/d (as-is)	301	301	301	0.8	0.998
g/d (DM basis)	278	279	279	0.8	0.985
Gross energy (MJ/d)	5.10	5.11	5.11	0.014	0.904
Nutrient intake					
OM	244	244	244	0.7	0.969
CP	70.4	70.8	71.0	0.20	0.161
NDF	63.4	63.3	63.2	0.17	0.836
Fecal output					
g/d (as-is)	276	274	272	4.7	0.851
g/d (DM basis)	89.7	88.5	85.8	1.26	0.142
Gross energy (MJ/d)	1.25	1.25	1.22	0.024	0.464
Fecal production (%)	32.0	31.8	30.8	0.42	0.134
Defecations (n/d)	2.4^a,b^	2.5^b^	2.3^a^	0.05	0.026
DM feces (%)	33.5	32.7	31.9	0.58	0.211
Fecal score (1–5)	3.4	3.4	3.5	0.04	0.208
ATTD					
DM	68.0	68.2	69.2	0.42	0.134
Organic matter	75.1	75.0	75.7	0.39	0.488
Crude protein	74.1	74.2	73.5	1.11	0.882
Neutral detergent fiber	48.6	48.3	51.0	0.72	0.058
Gross energy	75.5	75.4	76.3	0.45	0.305
Metabolizable energy	12.9	12.8	13.0	0.08	0.261
Fecal metabolites					
pH	6.76	6.72	6.68	0.066	0.704
Ammonia-N	2.57	2.47	2.40	0.200	0.837
Volatile fatty acids					
Total	1,219	1,259	1,250	90.8	0.948
Acetate	759	784	792	55.8	0.913
Propionate	321	333	314	34.7	0.928
*Iso*-butyrate	13.2	12.9	13.9	1.05	0.792
Butyrate	103	106	107	3.65	0.724
*Iso*-valerate	15.1	16.0	16.4	0.79	0.527
Valerate	3.38	4.32	3.01	0.402	0.116
*Iso*-caproate	2.97	2.96	3.64	0.606	0.671
Caproate	0.722	0.570	0.478	0.1111	0.342
Acetate:propionate	2.39	2.41	2.63	0.178	0.578

^a,b^Values with different superscript letters in the same row are significantly different (*p* < 0.05).

#### 3.3.2. Experiment 2. *Nannochloropsis oceanica*

Supplementation level of *N. oceanica* did not affect food and nutrient intake and fecal output (*p* > 0.05; Table 5). Despite the significant effect of *N. oceanica* inclusion level on fecal score (*p* = 0.036; Table 5), differences observed (3.3 vs. 3.4) lack biological meaning. Digestibility of DM, nutrients and energy, and ME content and fecal pH, and ammonia-N and VFA concentrations were not affected (*p* > 0.05) by level of supplementation with *N. oceanica*, except for *iso*-valerate (*p* = 0.051) and *iso*-caproate (*p* = 0.079) concentrations where a tendency was observed (Table 5).

**Table 5:** Experiment 2. Effects of increasing supplementation levels of *Nannochloropsis oceanica* on food, gross energy and nutrient intake (dry matter, DM, basis), fecal output and characteristics, apparent total tract digestibility (ATTD, %), metabolizable energy content (MJ kg^−1^ DM), and fecal pH, concentration of ammonia-N (g kg^−1^ DM), and concentration of volatile fatty acids (μmol g^−1^ DM).

	Diet			SEM	*p*-value
	0.5%	1.0%	1.5%		
Food intake					
g/d (as-is)	312	312	309	3.4	0.740
g/d (DM basis)	286	288	285	3.1	0.717
Gross energy (MJ/d)	5.23	5.28	5.21	0.058	0.706
Nutrient intake					
OM	249	251	248	2.7	0.686
CP	72.0	72.6	71.7	0.81	0.711
NDF	65.0	65.5	64.6	0.71	0.673
Fecal output					
g/d (as-is)	272	267	258	8.1	0.498
g/d (DM basis)	100.1	94.4	88.7	3.78	0.165
Gross energy (MJ/d)	1.31	1.31	1.28	0.032	0.794
Fecal production (%)	35.0	32.8	31.4	1.38	0.229
Defecations (n/d)	2.3	2.5	2.6	0.05	0.181
DM feces (%)	36.3	34.7	34.8	0.64	0.188
Fecal score (1–5)	3.4^a^	3.3^b^	3.3^b^	0.03	0.036
ATTD	65.0	67.2	68.6	1.38	0.233
DM	74.8	75.1	75.2	0.59	0.891
Organic matter	69.6	70.7	72.3	1.43	0.440
Crude protein	74.9	75.1	75.2	0.65	0.954
Neutral detergent fiber	49.6	49.0	49.4	1.37	0.943
Gross energy	65.0	67.2	68.6	1.38	0.233
Metabolizable energy	12.8	12.8	12.8	0.11	0.986
Fecal metabolites					
pH	6.72	6.81	6.85	0.085	0.573
Ammonia-N	2.96	2.58	2.93	0.287	0.608
Volatile fatty acids					
Total	1,391	1,338	1,282	73.5	0.597
Acetate	887	861	819	51.8	0.661
Propionate	364	350	331	16.3	0.401
*Iso*-butyrate	16.0	14.9	15.7	0.745	0.572
Butyrate	94.0	87.0	81.5	5.38	0.312
*Iso*-valerate	19.1	16.3	18.2	0.679	0.051
Valerate	4.43	4.03	9.51	1.928	0.142
*Iso*-caproate	4.52	3.34	4.79	0.411	0.079
Caproate	1.55	0.900	1.52	0.224	0.128
Acetate:propionate	2.44	2.46	2.46	0.072	0.958

^a,b^Values with different superscript letters in the same row are significantly different (*p* < 0.05).

#### 3.3.3. Experiment 3. *Tetradesmus obliquus*

Table 6 presents the effects of level of *T. obliquus* supplementation on food and nutrient intake, fecal output and characteristics, ATTD of DM, nutrients and energy and ME content, and fecal metabolites. Level of microalgae supplementation did not affect any of the parameters measured (*p* > 0.05) with the only exception of number of defecations that tended to be higher (*p* = 0.06) with 1.5% of supplementation.

**Table 6:** Experiment 3. Effects of increasing supplementation levels of *Tetradesmus obliquus* on food, gross energy and nutrient intake (dry matter, DM, basis), fecal output and characteristics, apparent total tract digestibility (ATTD, %), metabolizable energy content (MJ kg^−1^ DM), and fecal pH, concentration of ammonia-N (g kg^−1^ DM), and concentration of volatile fatty acids (μmol g^−1^ DM).

	Diet			SEM	*p*-value
	0.5%	1.0%	1.5%		
Food intake					
g/d (as-is)	301	301	301	0.02	0.130
g/d (DM basis)	278	277	279	0.9	0.450
Gross energy (MJ/d)	5.10	5.08	5.12	0.018	0.389
Nutrient intake					
Organic matter	243	242	244	0.8	0.445
Crude protein	70.4	70.2	70.9	0.25	0.181
Neutral detergent fiber	63.4	63.1	63.5	0.21	0.450
Fecal output					
g/d (as-is)	281	283	292	9.7	0.703
g/d (DM basis)	85.9	88.9	90.4	1.97	0.319
Gross energy (MJ/d)	1.21	1.27	1.29	0.036	0.362
Fecal production (%)	30.9	32.0	32.4	0.76	0.437
Defecations (n/d)	2.3	2.2	2.6	0.09	0.060
DM feces (%)	31.3	31.3	30.6	0.76	0.726
Fecal score (1–5)	3.2	3.0	3.0	0.11	0.397
ATTD					
DM	69.1	68.0	67.6	0.76	0.437
Organic matter	74.8	73.9	73.5	0.59	0.326
Crude protein	72.8	70.6	69.9	1.38	0.353
Neutral detergent fiber	48.5	47.6	47.9	1.12	0.857
Gross energy	76.1	75.1	74.9	0.74	0.493
Metabolizable energy	13.0	12.8	12.8	0.13	0.622
Fecal metabolites					
pH	6.72	6.75	6.77	0.050	0.709
Ammonia-N	2.51	2.54	2.57	0.112	0.940
Volatile fatty acids					
Total	847	837	855	11.7	0.571
Acetate	526	522	531	8.0	0.731
Propionate	218	209	224	7.75	0.419
*Iso*-butyrate	8.97	11.1	9.23	0.656	0.104
Butyrate	73.7	73.3	70.6	1.78	0.454
*Iso*-valerate	10.8	12.2	11.1	0.80	0.443
Valerate	2.82	2.64	2.15	0.398	0.504
*Iso*-caproate	5.93	4.84	4.78	0.527	0.279
Caproate	1.43	1.48	1.46	0.272	0.993
Acetate:propionate	2.43	2.53	2.41	0.108	0.706

### 3.4. Comparison between reference and experimental diets

Food and nutrient intake and fecal output from dogs fed diets supplemented with microalgae were not significantly different from the reference diet (*p* > 0.05; Table 7). Comparing to the reference diet, the dietary supplementation with *C. vulgaris* (*p* = 0.044) and *T. obliquus* (*p* = 0.035) decreased the number of defecations (Table 7). Consistency of feces of dogs fed the reference diet or diets with microalgae was classified as soft, shaped, and moist stools leaving spots on the floor (3.0) to approximately firm, shaped, and dry stools (3.5). Dry matter content of feces was higher with *N. oceanica* supplementation over the reference diet (35.5% vs. 32.0%; *p* = 0.010), no effect being observed with *T. obliquus* and *C. vulgaris* dietary inclusion (Table 7).

**Table 7:** Comparison between effects of reference diet and experimental diets supplemented with microalgae inclusion on food, gross energy and nutrient intake (dry matter, DM, basis), fecal output and characteristics, apparent total tract digestibility (ATTD, %), metabolizable energy content (MJ kg^−1^ DM), and fecal pH, concentration of ammonia-N (g kg^−1^ DM), and concentration of volatile fatty acids (μmol g^−1^ DM).

	Reference	*t*-test		
		*Chlorella vulgaris*	*Nannochloropsis oceanica*	*Tetradesmus obliquus*
Food intake				
g/d (as-is)	315 ± 39.8	0.938	0.914	0.984
g/d (DM basis)	291 ± 36.7	0.991	0.870	0.967
Gross energy (MJ/d)	5.33 ± 0.673	0.996	0.855	0.987
Nutrient intake				
OM	254 ± 32.1	0.994	0.832	0.969
CP	73.3 ± 9.26	0.919	0.864	0.958
NDF	66.3 ± 8.38	0.956	0.820	0.967
Fecal output				
g/d (as-is)	290 ± 4.64	0.724	0.351	0.938
g/d (DM basis)	90.7 ± 11.91	0.993	0.336	0.945
Gross energy (MJ/d)	1.31 ± 0.186	0.630	0.583	0.787
Fecal production (%)	31.2 ± 1.85	0.984	0.139	0.853
Defecations (n/d)	2.7 ± 0.31	0.044	0.104	0.035
DM feces (%)	32.0 ± 1.63	0.452	0.010	0.416
Fecal score (1–5)	3.4 ± 0.34	0.633	0.165	0.042
ATTD	68.7 ± 1.92	0.869	0.141	0.957
DM	74.9 ± 1.72	0.312	0.716	0.718
Organic matter	70.6 ± 3.35	0.012	0.541	0.471
Crude protein	75.3 ± 1.96	0.287	0.265	0.592
Neutral detergent fiber	47.9 ± 3.20	0.205	0.492	0.660
Gross energy	68.7 ± 1.92	0.869	0.141	0.957
Metabolizable energy	12.9 ± 0.32	0.408	0.224	0.553
Fecal metabolites				
pH	7.02 ± 0.182	0.007	0.019	0.005
Ammonia-N	2.54 ± 0.621	0.625	0.758	0.741
Volatile fatty acids				
Total	743 ± 104.5	<0.001	<0.001	0.061
Acetate	430 ± 74.0	<0.001	<0.001	0.003
Propionate	208 ± 28.2	0.001	<0.001	0.796
*Iso*-butyrate	10.1 ± 1.95	0.010	<0.001	0.567
Butyrate	74.8 ± 16.75	0.010	0.033	0.236
*Iso*-valerate	11.3 ± 1.60	<0.001	<0.001	0.780
Valerate	3.34 ± 1.695	0.928	0.231	0.073
*Iso*-caproate	4.39 ± 2.047	0.007	0.128	0.548
Caproate	1.06 ± 0.406	<0.001	<0.001	0.390
Acetate:propionate	2.07 ± 0.167	0.087	<0.001	0.026

Compared to the reference diet, the dietary supplementation with *T. obliquus* decreased the fecal score (3.1 vs. 3.4; *p* = 0.042), no changes being observed with *C. vulgaris* and *N. oceanica* (*p* > 0.05; Table 7). The reference diet showed an ATTD of DM and CP, respectively, of 68.7 and 70.6%, and a ME content of 12.9 MJ kg^−1^ DM (Table 7). Compared with the reference diet, the dietary inclusion of microalgae did not affect ATTD of DM, nutrients and energy, and ME content (*p* > 0.05), except for the inclusion of *C. vulgaris* that increased ATTD of CP (73.9% vs. 70.6%; *p* = 0.012; Table 7). Microalgae supplementation decreased fecal pH and increased acetate concentration, regardless of the microalgae species (Table 7). Supplementation with *C. vulgaris* and *N. oceanica* increased total VFA production and concentrations of propionate, *iso*-butyrate, butyrate and *iso*-valerate over the reference diet (*p* < 0.05; Table 7). Dietary supplementation with *T. obliquus* tended to increase (*p* = 0.061) total VFA production and increased acetate:propionate ratio (*p* = 0.026; Table 7). Caproate concentration decreased and increased with the inclusion of *C. vulgaris* and *N. oceanica*, respectively (Table 7). *Chlorella vulgaris* decreased (*p* < 0.001) *iso*-caproate concentration and tended to increase acetate:propionate ratio (*p* = 0.087; Table 7). Fecal ammonia-N concentration was not affected by microalgae supplementation (Table 7).

### 3.5. Fecal microbiota

**Figure 1:**
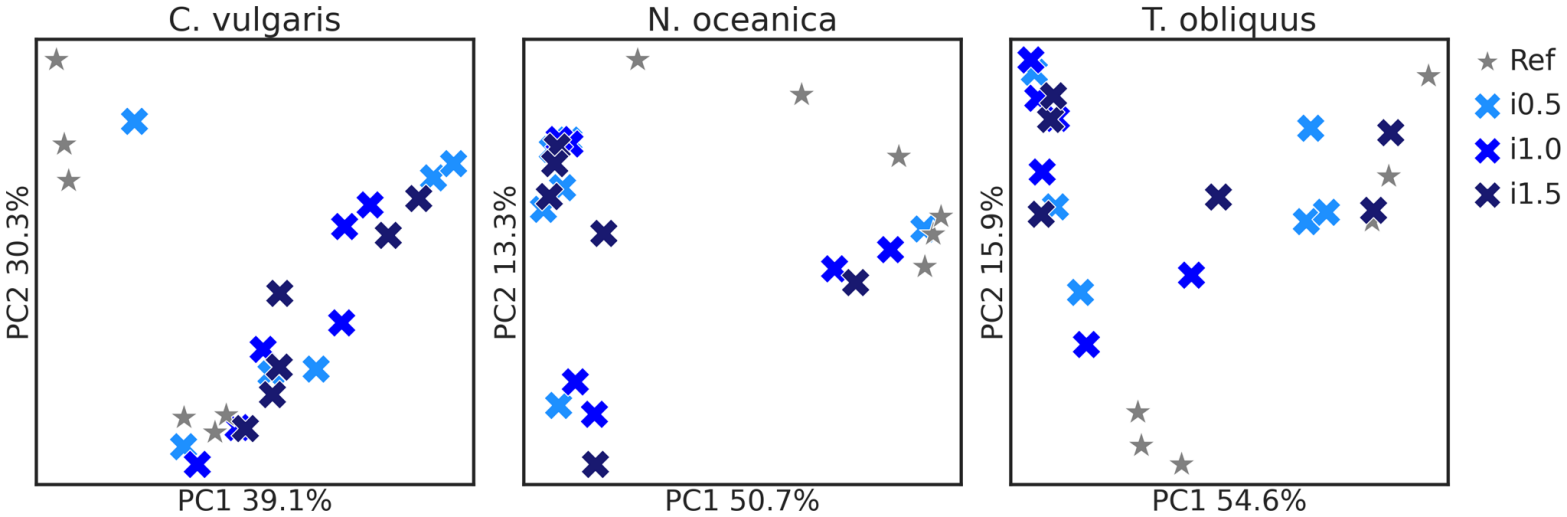
Principal-coordinate analysis plots of *Chlorella vulgaris*, *Nannochloropsis oceanica*, and *Tetradesmus obliquus* Bray–Curtis distances. Reference and supplemented diets differentiated by shapes and color and inclusion levels by color gradient.

Bacterial composition (Figure 1) was affected in studies with the supplementation of *C. vulgaris* (*p* = 0.043) and *N. oceanica* (*p* = 0.026), while in the study with *T. obliquus* only borderline significance was observed (*p* = 0.072). Differences between the reference diet and microalgae supplementation levels in the pairwise mode were significant for *N. oceanica* study (all *p* = 0.030) and borderline significant in study with *C. vulgaris* (all *p* = 0.050–0.051).

**Figure 2:**
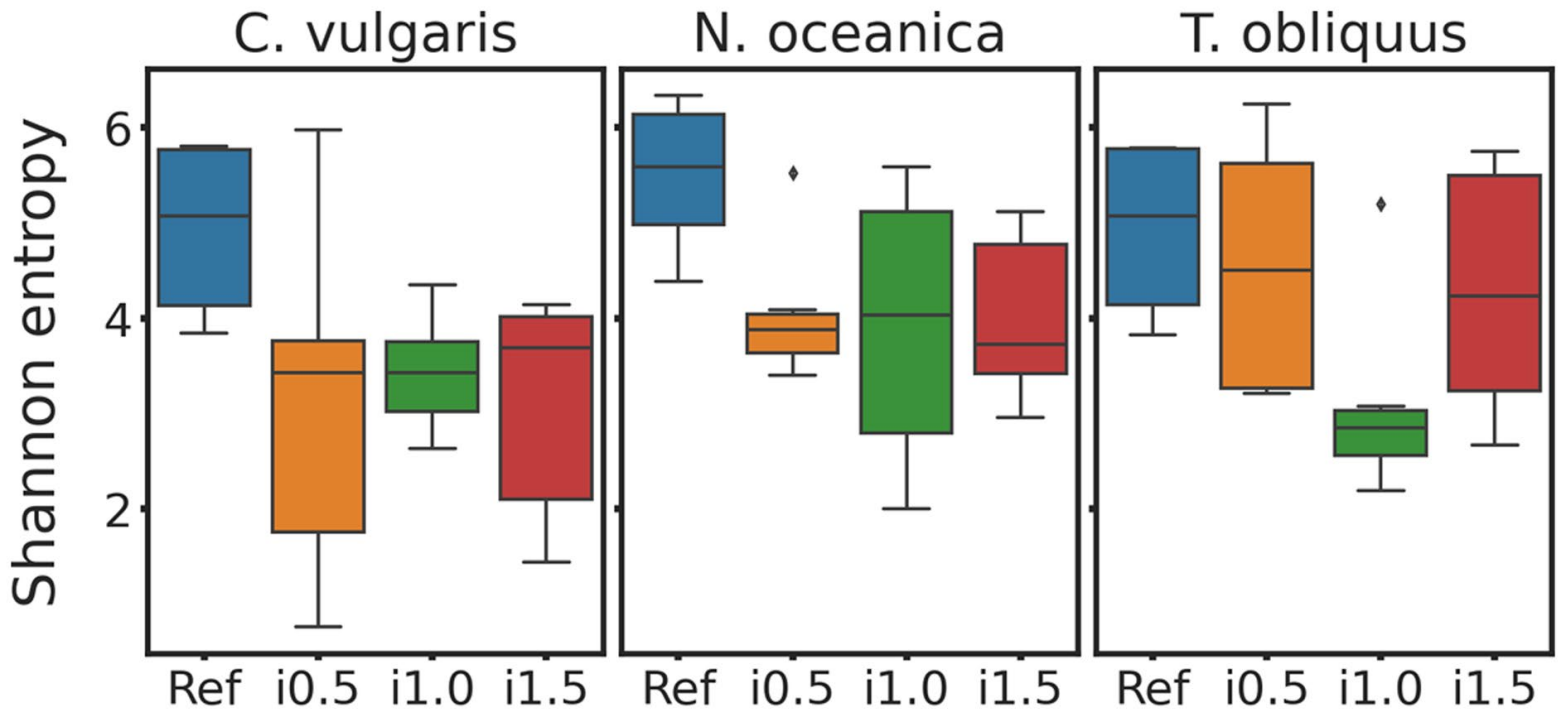
Boxplots of *Chlorella vulgaris*, *Nannochloropsis oceanica*, and *Tetradesmus obliquus* Shannon entropy. Reference and supplemented diets differentiated by color.

Regarding alpha diversity, some decrease in Shannon entropy (Figure 2) index was observed in boxplots of supplemented diets for all three studies, however, when tested with Wilcoxon test for dependent samples none of those differences turned out to be significant.

**Figure 3:**
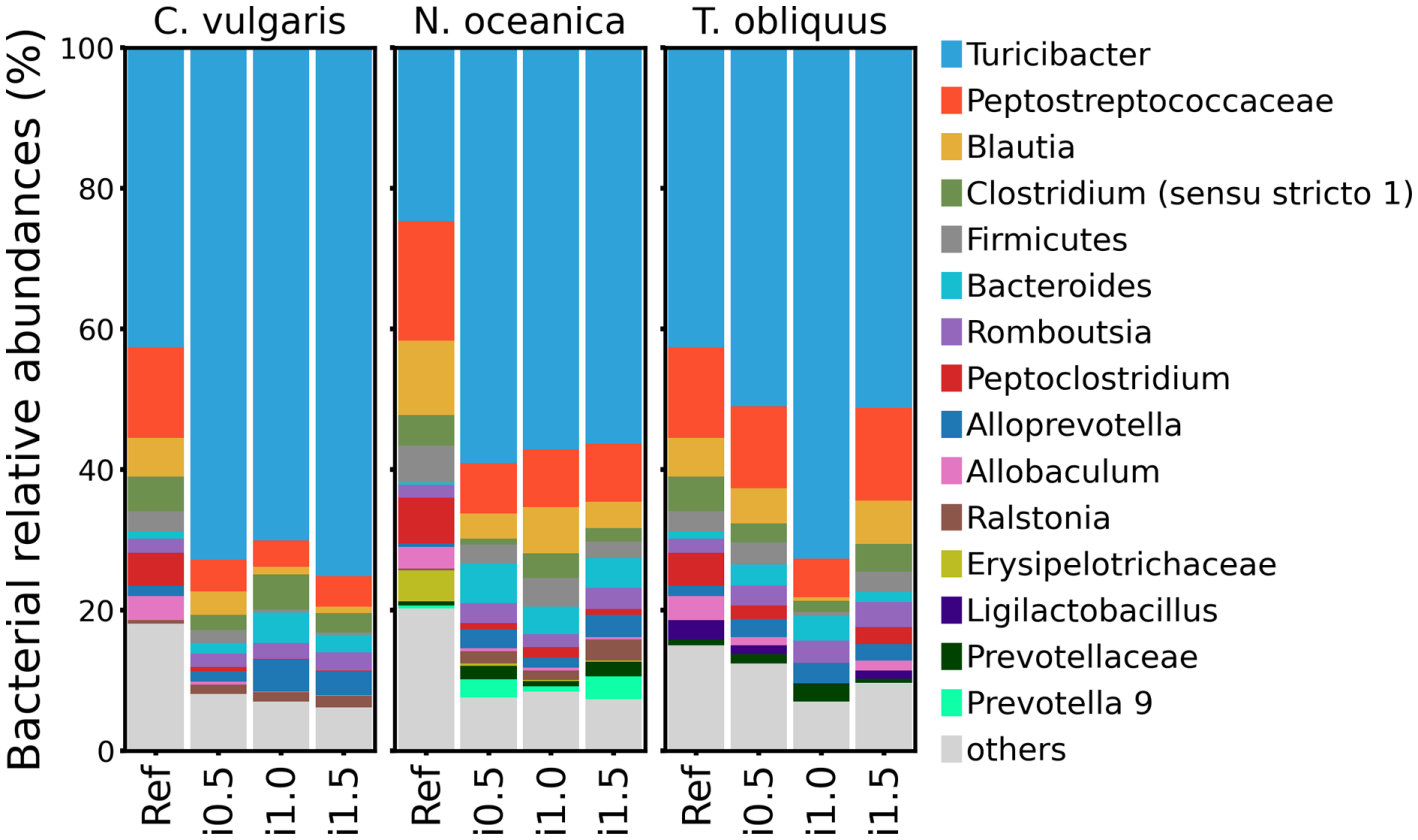
Taxonomy barplots of reference diet and diets with inclusion of *Chlorella vulgaris*, *Nannochloropsis oceanica*, and *Tetradesmus obliquus* at genus level or last available rank (if genus level was not assigned).

Among all studies, *Turicibacter* genus was consistently the most abundant, followed by unclassified Peptostreptococcaceae. *Blautia* was the third most abundant genus in *N. oceanica* and *T. obliquus* studies, but in the study with *C. vulgaris* supplementation, its abundance was outnumbered by *Clostridium* (*sensu stricto* 1) (Figure 3).

**Figure 4:**
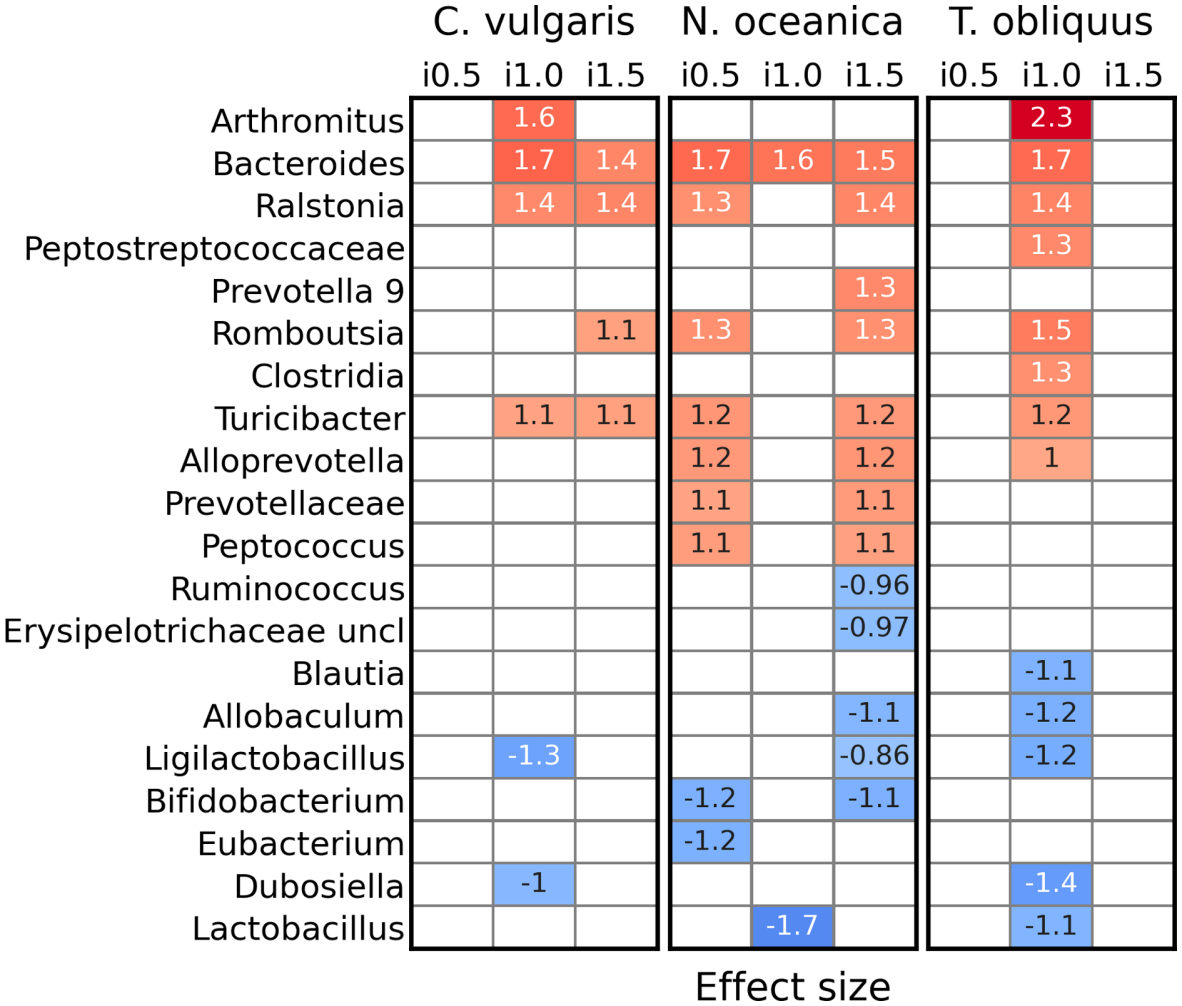
Differentially abundant genera (ALDEx2) between reference diet and diets with inclusion of *Chlorella vulgaris*, *Nannochloropsis oceanica*, and *Tetradesmus obliquus*.

Supplementation of *C. vulgaris* resulted in increased abundances (for inclusion levels 1 and 1.5%) of *Arthromitus*, *Bacteroides*, *Ralstonia*, *Romboutsia*, and *Turicibacter*, while abundances of *Ligilactobacillus* and *Dubosiella decreased* (Figure 4). In a study with *N. oceanica* its addition mostly affected bacterial abundances at inclusion levels 0.5 and 1.5%. So, counts of *Bacteroides*, *Ralstonia*, *Prevotella* 9, *Romboutsia*, *Turicibacter*, *Alloprevotella*, *Prevotellaceae*, and *Peptococcus* were increased and such of *Ruminococcus*, unclassified Erysipelotrichaceae, *Allobaculum*, *Ligilactobacillus*, *Bifidobacterium*, *Eubacterium*, and *Lactobacillus* decreased. The effect of the *T. obliquus* on bacterial genera was detected only at inclusion level 1%. Abundances of *Arthromitus*, *Bacteroides*, *Ralstonia*, unclassified Peptostreptococcaceae, *Clostridida*, *Romboutsia*, *Turicibacter*, and *Alloprevotella* increased, while representation of *Blautia*, *Allobaculum*, *Ligilactobacillus*, *Dubosiella*, and *Lactobacillus* went down. However, it should be noted that the significance of those differences between reference and supplemented diets were not dependent on the inclusion level itself. No differentially abundant genera were discovered between inclusion levels in all three studies.

## 4. Discussion

### 4.1. Palatability

Palatability is affected by several factors, such as flavor, food texture, size and shape of kibble, diet chemical composition [e.g., protein and fat contents; (47)], and intrinsic variables of the animals (48). Although first approach and taste were not significantly affected by 1.5% of dietary inclusion of the three microalgae species studied, the intake ratio was reduced with *C. vulgaris* and *N. oceanica*, with dogs preferring the reference diet. To the best of our knowledge, no studies evaluated the palatability of these microalgae species in dogs. However, Souza et al. (9) reported a positive effect of the dietary inclusion of 0.4% of microalgae *Schizochytrium* sp. in intake ratio and first choice, suggesting that the characteristic flavor (fishy smell) of the microalgae was responsible for the results obtained. Similarly, a recent study showed that dietary supplementation of *A. platensis* (0.05–0.19 g kg^−1^ BW) was well tolerated and accepted by dogs (7). Conversely, low palatability of microalgae of genera *Chlorella* and *Nannochloropsis* has been reported in fish and livestock (49, 50), and this lower acceptability has been associated with microalgae taste, odor, and physical structure of the dry powdery form (51). As palatability can be improved by changing the texture of the feed and adding palatants (52), the acceptability of dog diets with microalgae may be promoted if microalgae are included in the kibble, and not offered as-is as occurred in the present study.

### 4.2. Chemical composition

Dietary inclusion of the microalgae species studied up to 1.5% had minor effects on chemical composition of the experimental diets, that greatly reflected the composition of the reference diet and ensured the nutrient requirements established by FEDIAF (15) for adult dogs. Protein-rich microalgae species, like *C. vulgaris*, were proposed as an alternative protein ingredient to replace traditional human and animal feeding sources. Their high production cost and low cell wall digestibility have been overcome through the use of high yielding strains, optimization of culturing conditions, and pre-treatments of algal cells (53). In the present study, the dietary inclusion of *C. vulgaris* and *T. obliquus* suggested the potential to increase diet CP content and essential amino acids, with the only exceptions of His with *T. obliquus*. These results indicate that if included in higher levels in complete feeds, the microalgae studied can constitute useful protein sources to balance amino acids supply, namely lysine that is commonly the first limiting amino acid in diets including cereals and soybean (54). Additionally, the microalgae studied, particularly *C. vulgaris*, presented a higher content of individual essential amino acids, except histidine, than pet-food grade poultry by-product meal, a popular protein source in pet food (55).

Inclusion of *N. oceanica* can promote dietary lipids and eicosapentaenoic acid (EPA; C20:5 *n*-3) contents, decreasing the *n*-6/*n*-3 ratio. In commercial dog foods, the levels of individual fatty acids are quite variable and reflect different strategies of food producers in choosing lipid sources to ensure essential polyunsaturated fatty acids (PUFA) requirements (56). Higher levels of *n*-3 PUFA have been considered beneficial for animal health, namely for dogs with pruritus, renal insufficiency, and other inflammatory disorders, due to their anti-inflammatory properties (57, 58), particularly of the long-chain *n*-3 PUFA EPA and docosahexaenoic acid [DHA; C22:6 *n*-3; (59)]. Fish oil is the most common source of long-chain *n*-3 PUFA in commercial pet foods. However, the sustainability of this strategy is questionable as the high demand for fish oil for human and animal feeding is endangering fish stocks (60). In this context, *N. oceanica* can contribute to a more sustainable source of long-chain *n*-3 PUFA for dog feeding.

Microalgae are considered good sources of minerals. The species studied in the present study stands out for the high level of Na in *N. oceanica* and Fe in *T. obliquus* and *C. vulgaris* (8). Healthy dogs adjust to different dietary levels of Na through the rennin-angiotensin-aldosterone mechanisms, and no strong evidence is available on the risk of hypertension promoted by a high Na intake and on the ideal dietary Na level for dogs with cardiac deficiency (61). Moreover, increased dietary Na has been used as a dietary strategy to reduce the risk of urolithiasis (62). Storage of Fe in the organism is tightly regulated to ensure cellular needs without the development of toxicity and Fe homeostasis is controlled by the regulation of Fe efflux in the enterocytes through the hormone hepcidin (63). An excessive accumulation of Fe in hepatocytes can cause hemochromatosis, fibrosis and cirrhosis, whereas Fe deficiency can lead to anemia and other metabolic dysfunctions (64).

### 4.3. Food intake, fecal output and characteristics, *in vivo* digestibility, and metabolizable energy

Despite the commercial availability of some microalgae species, their use as ingredients in complete diets for dogs is limited, mainly by their still high production cost, being microalgae most commonly used as additives (in declared amounts lower than 0.5%) to take advantage of their functional value. From the results obtained in the palatability trials, the levels of microalgae supplementation to be studied in the digestibility trials were set at 0.5% (representing the most commonly used level in commercial foods and in scientific studies), 1.0, and 1.5%. Studies with other animal species found that a very low, economical acceptable level of dietary inclusion of microalgae biomass (0.1 to 1%) can benefit animal performance. Indeed, positive effects on mortality rate in mice (65), number of piglets alive, total weight of each litter and mortality rate prior and after weaning (66), and improved nutrient digestibility, feed intake and feed conversion in growing pigs (67) have been reported.

Microalgae supplementation up to 1.5% did not affect food intake, fecal output, digestibility of nutrients and energy and ME content, regardless of the species. Although information on digestibility and ME content of individual ingredients is valuable for diet formulation, studies on ingredient digestibility in dogs are scarce, and ME content is often estimated by chemical composition (15). To evaluate ingredient digestibility, the difference and the regression methods have been largely used in livestock (68, 69), but also applied to dogs (70, 71). The regression method requires the use of several experimental diets, to get information about the effects of different levels of inclusion, but it is more expensive. The generated equation is valid only for the range of the levels of ingredient inclusion used, whereas the difference method assumes no associative effects between the studied ingredient and the basal diet and requires a greater ingredient inclusion level to reduce estimative errors. These two methods can give conflicting results, as previous reported (72). In the present study, it was not possible to estimate digestibility of the three microalgae species studied as no linear regression was obtained between microalgae inclusion and diet digestibility, and the extrapolation to 100% microalgae inclusion provided digestibility values higher than 100%. Similarly, Kawauchi et al. (72) could not determine all nutrients digestibility by the regression and difference methods. No studies evaluating the digestibility of the three microalgae species studied were found in the literature.

Digestibility and consequent fecal output indicate diet quality and assume relevant importance to pet owners from a waste disposal perspective. The commercial complete food used as the reference diet includes cereals and animal meals as main ingredients (label information) and presented modest ATTD of OM, CP, and energy. Digestibility is known to be affected by several factors such as chemical composition, processing methods, and the physiological state of the animal, and depending on the water holding capacity, higher nutrient digestibility usually results in lower fecal output. The digestibility of microalgae, particularly in monogastric animals, is mainly constrained by their rigid cellulosic cell wall that limits the access to the cell contents. Therefore, processing methods of microalgae to disrupt the cell wall have been proposed (53), with reported benefits on growth, feed conversion (73), and digestibility (74). In the present study, dogs were fed whole microalgae without previous processing, thus, higher digestibility might be expected if disrupted microalgae are used.

Number of defecations decreased with *C. vulgaris* and *T. obliquus* inclusion over the reference diet but were not significantly affected by microalgae level. Dietary contents of soluble and insoluble fiber and ash (72, 75), have been suggested to affect frequency of defecation, but results are contradictory. Indeed, El-Wahab et al. (76) reported no differences in the frequency of defecation (1.86–2.29) between vegetarian diets with an ash content of 41.3 to 53.8 g/kg DM. Similarly, Ingenpaß et al. (77) observed no effect on frequency of defecation (2.30–2.57) between meat and vegetarian-based diets with an ash content of 62.4 and 55.1 g/kg DM, respectively. In the present study, soluble and insoluble dietary fiber was not analyzed, and dietary ash content was very similar among diets. However, the high ash content of *N. oceanica* might affect the frequency of defecation if higher inclusion levels are used.

Along with frequency of defecation, dog owners judge the quality of a commercial feed based on fecal quality. Compared to the reference diet, inclusion of *N. oceanica* increased feces DM content, and *T. obliquus* decreased fecal scores. Fecal DM content and fecal consistency scores are used to determine fecal consistency (78), but an association between these two parameters is not always observed (77). Increased proteolytic fermentation in the hindgut and diet supplementation with non-digestible oligosaccharides are known to decrease fecal DM content (79). Indeed, indigestible protein provide a substrate for fermentation by proteolytic bacteria, promoting the osmotic pressure, thus greater water to the intestinal lumen and reducing fecal quality (46). Similarly, end-products of fiber fermentation in the large intestine increase the osmotic pressure in the intestinal lumen. The high-water holding capacity of soluble fibers decreases fecal DM content (80). However, microalgae inclusion effects on dietary fiber digestibility and colonic fermentation products do not support differences between experimental diets on extent of protein and fiber fermentation in the large intestine.

#### 4.3.1. Fecal pH, ammonia-N, and volatile fatty acids concentrations

Comparing to the reference diet, the dietary inclusion of the three microalgae species studied decreased fecal pH, suggesting a reduced colonic fermentation of nitrogen compounds, as it is known that the fermentation of non-digested protein produces nitrogen compounds, such as ammonia-N and branched-chain VFA that increases intestinal pH and might harm intestinal health and worse fecal odor (81). However, no effects of microalgae inclusion have been observed on fecal ammonia-N concentration and *C. vulgaris* and *N. oceanica* increased branched-chain VFA concentrations. These results suggest that the decrease in feces pH was not mainly driven by effects on the amount of indigestible protein in the colon, but rather due to total VFA production.

Dietary inclusion of all microalgae species studied increased total VFA (*p* = 0.061 for *T. obliquus*). Several factors are known to affect VFA production, such as substrate source for colonic fermentation, gastrointestinal transit time and microbiota composition. VFA comprise a source of energy for bacterial metabolism, growth of epithelial cells, and for the host animal providing up to 7% of the maintenance energy requirements of an adult dog (82). Additionally, VFA regulates luminal pH and mucus secretion and provides some health-promoting effects, namely anti-inflammatory, immunomodulatory, and anticarcinogenic (83).

Acetate, propionate and butyrate are the most abundant VFA in dog feces, comprising approximately 60, 25, and 10% of the total VFA (84). Fecal acetate concentration increased with the dietary inclusion of all microalgae studied and *C. vulgaris* and *N. oceanica* inclusion also increased the concentrations of the other measured VFA except for valerate and iso-caproate (for *N. oceanica*). Acetate is the most abundant VFA in the colon and most bacteria produce acetate from carbohydrate fermentation. It is mainly metabolized by peripheral tissues, thus, high concentrations reach the systemic circulation, and depending on the tissues is involved in the citric acid cycle or fatty acid synthesis (85). Similarly to acetate, the majority of the propionate produced enters the portal circulation and is metabolized in the liver. Propionate is used for glucose synthesis, contributing to a reduction of blood sugar and serum cholesterol, and exerts anti-inflammatory effects in the intestine, decreasing the production of pro-inflammatory cytokines such as IL-6, IL-8, and TNFα (86). As fecal concentration of propionate is decreased in dogs with gastrointestinal diseases, it comprises a biomarker of gut functionality in dogs (87). Only small amounts (<10%) of butyrate reaches the portal circulation, being butyrate the main energy source for colonocytes, also plays a role in maintaining cell growth and differentiation in the gut, preventing colonic cancer and reducing inflammation (85).

Branched-chain VFA represent minor components of the VFA and result from the bacterial degradation of the branched-chain amino acids valine, leucine and isoleucine (88). The increase of branched-chain VFA observed in the present study with the dietary inclusion of *C. vulgaris* and *N. oceanica* suggests an increased proteolytic activity of some bacterial populations.

### 4.4. Fecal microbiota

It is well known that diet can exert a strong influence on gastrointestinal health, fecal microbiota, and fecal metabolite concentrations (89, 90). Most studies comprise a dietary period of 2 to 4 weeks before sampling feces to allow the stabilization of the microbial community and activity (91). However, the longitudinal changes promoted by a dietary change in gut microbial phylogeny and function, and metabolite profiles have not been well studied in dogs. Recently, Lin et al. (92) studied the kinetics required by a dietary change from a control diet to a fiber supplemented diet or a protein-rich canned diet to modify the fecal microbiome and metabolites in healthy adult Beagle dogs and found that fecal microbial diversity, composition and function as well as metabolite profiles (pH, VFA, ammonia-N) dramatically changed and stabilized within a few days (2 d for metabolites and 6 d for microbiota) after dietary changes. In the present study, dogs were fed each diet for 10 days, and unlike previous research (93), microalgae supplementation affected the microbial composition of canine feces. Thus, abundances of *Turicibacter* and *Peptococcus* genera, which were shown to be correlated with butyrate (94, 95), increased in the feces of dogs on microalgae-supplemented diets. A similar enhancement of *Turicibacter* abundances in response to algae supplementation was reported in rats (96). Our study observed that dogs fed on diets with microalgae supplementation decreased the number of defecations. In a comparative study of healthy dogs and dogs with chronic diarrhea, *Turicibacter* abundances were shown to be correlated with the healthy group, suggesting its protective role in gut health (97, 98). Other genera, which abundances increased in dogs fed microalgae-supplemented diets were *Bacteroides*, *Peptococcus*, *Romboutsia*, *Prevotella* 9, *Alloprevotella*, *Arthromitus*, and some unassigned to genus level Prevotellaceae, Clostridia, and Peptostreptococcaceae. Members of those taxa are often associated with elevated VFA content (99–102), which agrees with the observed increased amount of total VFA production in feces of dogs fed with microalgae-supplemented diets. Moreover, *Bacteroides* and *Prevotella* spp. are involved in the degradation of complex plant polysaccharides (103–105) and their increased content may be a direct consequence of algae supplementation. *Bacteroides* ability to utilize urea as a nitrogen source (106) also corresponds with lower feces pH under supplemented diets. *Arthromitus* spp. are not only associated with VFA production, but also related to the activation of the immune system (107). *Peptococcus* abundances were reported to be positively correlated with fecal butyrate and likewise associated with dogs’ gut health (95). More genera whose growth was stimulated by algae supplementation, *Romboutsia* and *Alloprevotella*, were shown to play an important role in dogs’s health via higher carbohydrate utilization (90, 108) and improvements in body-weight regulation (109), respectively. Interestingly, some bacterial taxa that were in lower abundance with algae supplementation, such as *Blautia*, *Allobaculum*, and *Ruminococcus* were previously reported to be essential players in dogs weight regulation (110, 111). In our study, *Turicibacter* genus average abundances prevailed in all diets, including the reference. However, as is shown in the Supplementary Figure S1, feces microbiome varied between different dogs, and despite the average dominance, the *Turicibacter* genus was not the most abundant one in some dogs, supporting interindividual variability (112) between even adult dogs. Relatively high abundances of the *Turicibacter* genus in the reference diet compared to the other studies (113, 114) suggest that in our study either housing conditions or targeted 16S rRNA region (V1–V2) were responsible for the *Turicibacter* dominance.

## 5. Conclusion

The present study shows that dietary inclusion of *C. vulgaris*, *N. oceanica*, and *T. obliquus* up to 1.5% had no negative effect on chemical composition, food intake and digestibility of diets, but the acceptability of diets for dogs with microalgae can be favored if the microalgae are included in the kibbles. In addition, feeding dogs with microalgae-supplemented diets affected fecal characteristics and altered microbiota composition towards the promotion of *Turicibacter* and *Peptococcus* genera associated with gut health and activation of the immune system. Overall, the results support the potential of *T. obliquus*, *C. vulgaris*, and *N. oceanica* as sustainable functional additives for dog feeding.

## Data availability statement

The datasets presented in this study can be found in online repositories. The names of the repository/repositories and accession number(s) can be found below: [https://www.ebi.ac.uk/ena AND PRJEB61064].

## Ethics statement

The animal study was approved by Animal Ethics Committee of School of Medicine and Biomedical Sciences, University of Porto. The study was conducted in accordance with the local legislation and institutional requirements.

## Author contributions

AC designed the experiment, analyzed the data, obtained funding, drafted the original manuscript, and elaborated the final version of the manuscript. JG-F performed the experimental protocols. MS performed the experimental protocols. MM designed the experiment, participated in the data analysis, and critically revised the manuscript. TY performed the microbiota analysis and drafted the original manuscript. AC-S performed the microbiota analysis and critically revised the manuscript. AF designed the experiment, obtained funding, critically revised the manuscript, and elaborated the final version of the manuscript. All authors contributed to the article and approved the submitted version.

## Funding

This research was funded by the Portuguese Foundation for Science and Technology (FCT/MCTES) through projects UIDB/50006/2020 and UIDP/50006/2020, and from projects NovInDog (POCI-01-0247-FEDER-047003) and AlgaValor (POCI-01-0247-FEDER-035234; Lisboa-01-0247-FEDER-035234; ALG-01-0247-FEDER-035234) supported by Portugal 2020 program through the European Regional Development Fund. JG-F was funded by FCT and Soja de Portugal (PD/BDE/150527/2019), and MRGM by FCT through program DL 57/2016 – Norma transitória (SFRH/BPD/70176/2010).

## Conflict of interest

The authors declare that the research was conducted in the absence of any commercial or financial relationships that could be construed as a potential conflict of interest.

## Publisher’s note

All claims expressed in this article are solely those of the authors and do not necessarily represent those of their affiliated organizations, or those of the publisher, the editors and the reviewers. Any product that may be evaluated in this article, or claim that may be made by its manufacturer, is not guaranteed or endorsed by the publisher.

## Footnotes

^1^ https://github.com/najoshi/sabre
