# Supplementary material for: Effects of microalgae as dietary supplement on palatability, digestibility, fecal metabolites, and microbiota in healthy dogs
Source: Front Vet Sci. 2023 Sep 27;10:1245790. doi: 10.3389/fvets.2023.1245790 (PMC10565105; doi:10.3389/fvets.2023.1245790)
Supplement: Supplementary file 1 [file Data_Sheet_1.PDF]

Effects of microalgae as dietary supplement on palatability, digestibility, fecal metabolites and microbiota in healthy dogs

Ana R. J. Cabrita[1*], Joana Guilherme-Fernandes[1], Maria Spínola[1†], Margarida R. G. Maia[1], Timur Yergaliyev[2,3], Amélia Camarinha-Silva[2,3], António J. M. Fonseca[1]

*** Correspondence:** arcabrita@icbas.up.pt

Table S1. Proximate composition (g kg^{-1} dry matter, DM), gross energy (MJ kg^{-1} DM), amino acids, macro- and trace elements, and fatty acids content (g kg^{-1} DM) of the microalgae species.

	**Microalgae**		
	Chlorella vulgaris	***Nannochloropsis oceanica***	***Tetradesmus obliquus***
**Proximate composition**			
**DM, g/kg**	978	988	982
**Ash**	98.9	340	110
**Crude protein**	439	246	411
**Total lipids**	97.9	140	83.8
**Neutral detergent fiber**	164	153	228
**Acid detergent fiber**	97.8	43.4	119
**Starch**	44.1	0.693	10.7
**Gross energy**	20.3	16.6	21.5
**Essential amino acids**			
**Arginine**	46.6	22.1	27.2
**Histidine**	9.51	5.19	4.61
**Lysine**	53.2	21.9	25.3
**Threonine**	28.0	14.5	21.7
**Isoleucine**	21.8	11.3	15.7
**Leucine**	39.7	20.7	28.4
**Valine**	29.1	14.8	20.2
**Methionine**	10.9	5.34	7.67
**Methionine + cystine**	12.8	6.24	9.16
**Phenylalanine**	29.9	14.3	21.3

Table S1 (cont.). Proximate composition (g kg^{-1} dry matter, DM), gross energy (MJ kg^{-1} DM), amino acids, macro- and trace elements, and fatty acids content (g kg^{-1} DM) of the microalgae species.

	**Microalgae**		
	Chlorella vulgaris	***Nannochloropsis oceanica***	***Tetradesmus obliquus***
**Phenylalanine + tyrosine**	57.4	27.2	38.9
**Non-essential amino acids**			
**Cystine**	1.94	0.90	1.49
**Tyrosine**	27.6	12.8	17.6
**Aspartic acid + Asparagine**	34.9	21.2	31.2
**Glutamic acid + Glutamine**	52.3	31.3	41.8
**Alanine**	35.6	17.7	28.6
**Glycine**	39.2	18.9	27.0
**Proline**	25.5	15.6	19.1
**Serine**	24.8	13.3	18.9
**Macro elements**			
**Na**	0.500	37.8	1.80
**K**	8.50	19.1	12.8
**Mg**	0.560	3.91	0.540
**Ca**	4.64	4.36	4.36
**P**	25.7	18.2	18.2
**Ca:P ratio**	0.181	0.239	0.239
**Trace elements (mg kg^{-1} DM)**			
**Fe**	644	300	2986
**Mn**	163	35.2	108
**Cu**	25.4	13.6	7.22
**Zn**	357	52.5	113
**Se**	0.170	1.36	0.310
**Fatty acids**			
**C8:0**	0.015	0.074	0.006
**C10:0**	0.073	0.123	0.089
**C12:0**	0.127	0.340	0.065
**C14:0**	1.21	4.79	1.51
**C14:1 *n*-5**	0.010	0.057	0.019

Table S1 (cont.). Proximate composition (g kg^{-1} dry matter, DM), gross energy (MJ kg^{-1} DM), amino acids, macro- and trace elements, and fatty acids content (g kg^{-1} DM) of the microalgae species.

	**Microalgae**		
	Chlorella vulgaris	***Nannochloropsis oceanica***	***Tetradesmus obliquus***
***iso*-C15:0**	0.671	0.388	0.254
***anteiso*-C15:0**	0.163	0.059	0.069
**C15:0**	0.114	0.281	0.221
***iso*-C16:0**	0.128	0.045	0.045
**C16:0**	11.8	18.8	11.7
**C16:1 *n*-7**	1.21	21.5	1.12
**C16:1 *n*-9**	2.03	1.02	2.53
**C16:2 *n*-4**	0.088	0.149	0.057
**C16:3 *n*-4**	0.022	0.174	0.030
**C16:4 *n*-1**	0.176	0.060	0.050
***iso*-C17:0**	2.80	0.716	2.57
**C17:0**	0.330	0.181	0.308
**C17:1 *n*-8**	0.096	0.115	1.47
**C18:0**	1.77	0.343	0.435
**C18:1 *n*-7**	1.36	0.518	1.45
**C18:1 *n*-9**	2.13	3.82	3.59
**C18:2 *n*-6**	10.4	2.77	3.69
**C18:2 t*n*-6**	0.083	0.038	0.157
**C18:3 *n*-3**	18.4	0.227	18.6
**C18:3 *n*-6**	0.045	0.263	0.256
**C18:4 *n*-3**	0.057	0.077	2.01
**C20:0**	0.016	0.031	0.040
**C20:1 *n*-7**	0.012	0.005	0.015
**C20:1 *n*-9**	0.035	0.043	0.068
**C20:1 *n*-11**	0.013	0.018	0.041
**C20:2 *n*-6**	0.055	0.058	0.016
**C20:3 *n*-3**	0.016	0.091	0.325
**C20:4 *n*-3**	0.020	0.045	BDL

Table S1 (cont.). Proximate composition (g kg^{-1} dry matter, DM), gross energy (MJ kg^{-1} DM), amino acids, macro- and trace elements, and fatty acids content (g kg^{-1} DM) of the microalgae species.

	**Microalgae**		
	Chlorella vulgaris	***Nannochloropsis oceanica***	***Tetradesmus obliquus***
**C20:4 *n*-6**	0.020	0.045	BDL
**C20:5 n-3 (EPA)**	0.106	17.8	0.169
**C21:5 *n*-3**	0.079	0.193	0.059
**C22:0**	0.096	0.078	0.227
**C22:1 *n*-11**	0.045	0.026	0.044
**C22:2 *n*-6**	0.010	0.017	0.014
**C22:4 *n*-6**	BDL	BDL	BDL
**C22:5 *n*-3**	BDL	0.023	BDL
**C22:6 *n*-3 (DHA)**	BDL	BDL	0.195
**C24:0**	0.199	0.050	0.235
**C24:1 *n*-9**	0.020	BDL	0.262
**Sums and ratio**			
**SFA**	16.7	25.9	16.5
**MUFA**	7.01	27.1	9.19
**PUFA**	32.8	27.5	28.9
**PUFA *n*-3**	18.7	18.5	21.3
**PUFA *n*-6**	10.8	8.01	4.24
***n*-6/*n*-3 ratio**	0.579	0.434	0.199

BDL: below the detection limit; SFA: sum of saturated fatty acids; MUFA: sum of monounsaturated fatty acids; PUFA: sum of polyunsaturated fatty acids; *n*-6/*n*-3 ratio: ratio of PUFA *n*-6 to PUFA *n*-3.

Table S2. Content of non-essential amino acids and fatty acids content (g kg^{-1} dry matter) of the reference and experimental diets with inclusion of microalgae in substitution of the reference diet.

	**Diet**									
	**Reference**	***Chlorella vulgaris***			***Nannochloropsis oceanica***			***Tetradesmus obliquus***		
		**0.5%**	**1.0%**	**1.5%**	**0.5%**	**1.0%**	**1.5%**	**0.5%**	**1.0%**	**1.5%**
**Non-essential amino acids**										
**Cystine**	4.56	4.55	4.53	4.52	4.54	4.52	4.51	4.54	4.53	4.51
**Tyrosine**	6.82	6.92	7.03	7.13	6.85	6.88	6.91	6.87	6.93	6.98
**Aspartic acid + Asparagine**	21.8	21.9	21.9	22.0	21.8	21.8	21.8	21.9	21.9	21.9
**Glutamic acid + Glutamine**	39.9	40.0	40.0	40.1	39.9	39.8	39.8	39.9	39. 9	39.9
**Alanine**	19.4	19.5	19.6	19.6	19.4	19.4	19.4	19.5	19.5	19.5
**Glycine**	26.7	26.8	26.8	26.9	26.7	26.6	26.6	26.7	26.7	26.7
**Proline**	24.8	24.8	24.8	24.8	24.8	24.7	24.7	24.8	24.7	24.7
**Serine**	15.5	15.5	15.6	15.6	15.5	15.5	15.5	15.5	15.5	15.6
**Fatty acids**										
**C8:0**	0.016	0.016	0.016	0.016	0.016	0.017	0.017	0.016	0.016	0.016
**C10:0**	0.082	0.082	0.082	0.082	0.082	0.083	0.083	0.082	0.082	0.082
**C12:0**	0.097	0.097	0.097	0.097	0.098	0.099	0.101	0.097	0.097	0.096
**C14:0**	0.910	0.912	0.913	0.915	0.930	0.949	0.969	0.913	0.916	0.919
**C14:1 *n*-5**	0.100	0.099	0.099	0.098	0.099	0.099	0.099	0.099	0.099	0.098
***iso*-C15:0**	0.023	0.026	0.029	0.032	0.024	0.026	0.028	0.024	0.025	0.026
***anteiso*-C15:0**	0.034	0.035	0.035	0.036	0.034	0.034	0.034	0.034	0.034	0.034

Table S2 (cont.). Content of non-essential amino acids and fatty acids content (g kg^{-1} dry matter) of the reference and experimental diets with inclusion of microalgae in substitution of the reference diet.

	**Diet**												
	**Reference**		***Chlorella vulgaris***				***Nannochloropsis oceanica***				***Tetradesmus obliquus***		
			**0.5%**	**1.0%**	**1.5%**		**0.5%**	**1.0%**	**1.5%**		**0.5%**	**1.0%**	**1.5%**
**C15:0**	0.127		0.127	0.127	0.127		0.128	0.128	0.129		0.127	0.128	0.128
***iso*-C16:0**	0.027		0.027	0.028	0.029		0.027	0.027	0.027		0.027	0.027	0.027
**C16:1 *n*-9**	0.288		0.297	0.305	0.314		0.292	0.295	0.299		0.299	0.310	0.322
**C16:2 *n*-4**	BDL		0.000	0.001	0.001		0.001	0.001	0.002		0.000	0.001	0.001
**C16:3 *n*-4**	BDL		0.000	0.000	0.000		0.001	0.002	0.003		0.000	0.000	0.000
**C16:4 *n*-1**	0.026		0.027	0.028	0.028		0.026	0.027	0.027		0.026	0.026	0.027
***iso*-C17:0**	0.091		0.091	0.092	0.092		0.091	0.091	0.090		0.093	0.094	0.096
**C17:0**	0.314		0.314	0.314	0.314		0.313	0.312	0.312		0.314	0.314	0.314
**C17:1 *n*-8**	BDL		0.000	0.001	0.001		0.001	0.001	0.002		0.007	0.015	0.022
**C18:1 *n*-7**	2.02		2.01	2.01	2.01		2.01	2.00	1.99		2.01	2.01	2.01
**C18:2 t*n*-6**	0.070		0.070	0.070	0.070		0.070	0.069	0.069		0.070	0.071	0.071
**C18:3 *n*-6**	0.070		0.070	0.070	0.069		0.071	0.072	0.073		0.071	0.072	0.073
**C18:4 *n*-3**	0.023		0.023	0.023	0.023		0.023	0.023	0.023		0.033	0.043	0.052
**C20:0**	0.210		0.209	0.208	0.207		0.209	0.208	0.208		0.209	0.209	0.208
**C20:1 *n*-7**	0.022		0.022	0.022	0.022		0.022	0.022	0.022		0.022	0.022	0.022
**C20:1 *n*-9**	0.449		0.447	0.445	0.443		0.447	0.445	0.443		0.447	0.445	0.443
**C20:1 *n*-11**	0.160		0.159	0.158	0.157		0.159	0.158	0.157		0.159	0.158	0.158
**C20:2 n-6**	0.222		0.221	0.220	0.220		0.221	0.220	0.220		0.221	0.220	0.219

Table S2 (cont.). Content of non-essential amino acids and fatty acids content (g kg^{-1} dry matter) of the reference and experimental diets with inclusion of microalgae in substitution of the reference diet.

	Diet												
	**Reference**		***Chlorella vulgaris***				***Nannochloropsis oceanica***				***Tetradesmus obliquus***		
			**0.5%**	**1.0%**	**1.5%**		**0.5%**	**1.0%**	**1.5%**		**0.5%**	**1.0%**	**1.5%**
**C20:3 *n*-3**	0.028		0.027	0.027	0.027		0.028	0.028	0.028		0.029	0.030	0.032
**C20:4 *n*-3**	0.024		0.024	0.024	0.024		0.024	0.024	0.024		0.023	0.023	0.023
**C21:5 *n*-3**	0.023		0.024	0.024	0.024		0.024	0.025	0.026		0.024	0.024	0.024
**C22:0**	0.114		0.114	0.114	0.114		0.114	0.114	0.114		0.115	0.115	0.116
**C22:1 *n*-11**	0.099		0.099	0.099	0.099		0.099	0.099	0.098		0.099	0.099	0.099
**C22:2 *n*-6**	0.006		0.006	0.006	0.006		0.006	0.006	0.007		0.006	0.006	0.006
**C22:4 *n*-6**	0.098		0.097	0.097	0.096		0.097	0.097	0.096		0.097	0.097	0.096
**C22:5 *n*-3**	0.054		0.054	0.054	0.053		0.054	0.054	0.054		0.054	0.054	0.053
**C24:0**	0.122		0.123	0.123	0.123		0.122	0.122	0.121		0.123	0.123	0.124
**C24:1 *n*-9**	0.064		0.064	0.063	0.063		0.063	0.063	0.063		0.065	0.066	0.067
**Sums and ratio**													
**SFA**	30.1		30.1	30.0	29.9		30.1	30.1	30.1		30.1	30.0	29.9
**MUFA**	35.0		34.8	34.7	34.6		34.9	34.9	34.9		34.9	34.7	34.6
**PUFA**	24.8		24.8	24.9	24.9		24.8	24.8	24.8		24.8	24.8	24.8
**PUFA *n*-3**	1.38		1.47	1.55	1.64		1.46	1.55	1.64		1.48	1.58	1.68
**PUFA *n*-6**	23.0		23.0	22.9	22.8		22.9	22.9	22.8		22.9	22.8	22.7
***n*-6/*n*-3 ratio**	16.7		16.6	16.5	16.5		16.6	16.5	16.5		16.6	16.5	16.4

BDL: below the detection limit; SFA: sum of saturated fatty acids; MUFA: sum of monounsaturated fatty acids; PUFA: sum of polyunsaturated fatty acids; *n*-6/*n*-3 ratio: ratio of PUFA *n*-6 to PUFA *n*-3.

Figure S1. Taxonomy barplots of reference diet and diets with the inclusion of *Chlorella vulgaris*, *Nannochloropsis oceanica*, and *Tetradesmus obliquus* at genus level or last available rank (if genus level was not assigned) by dogs.

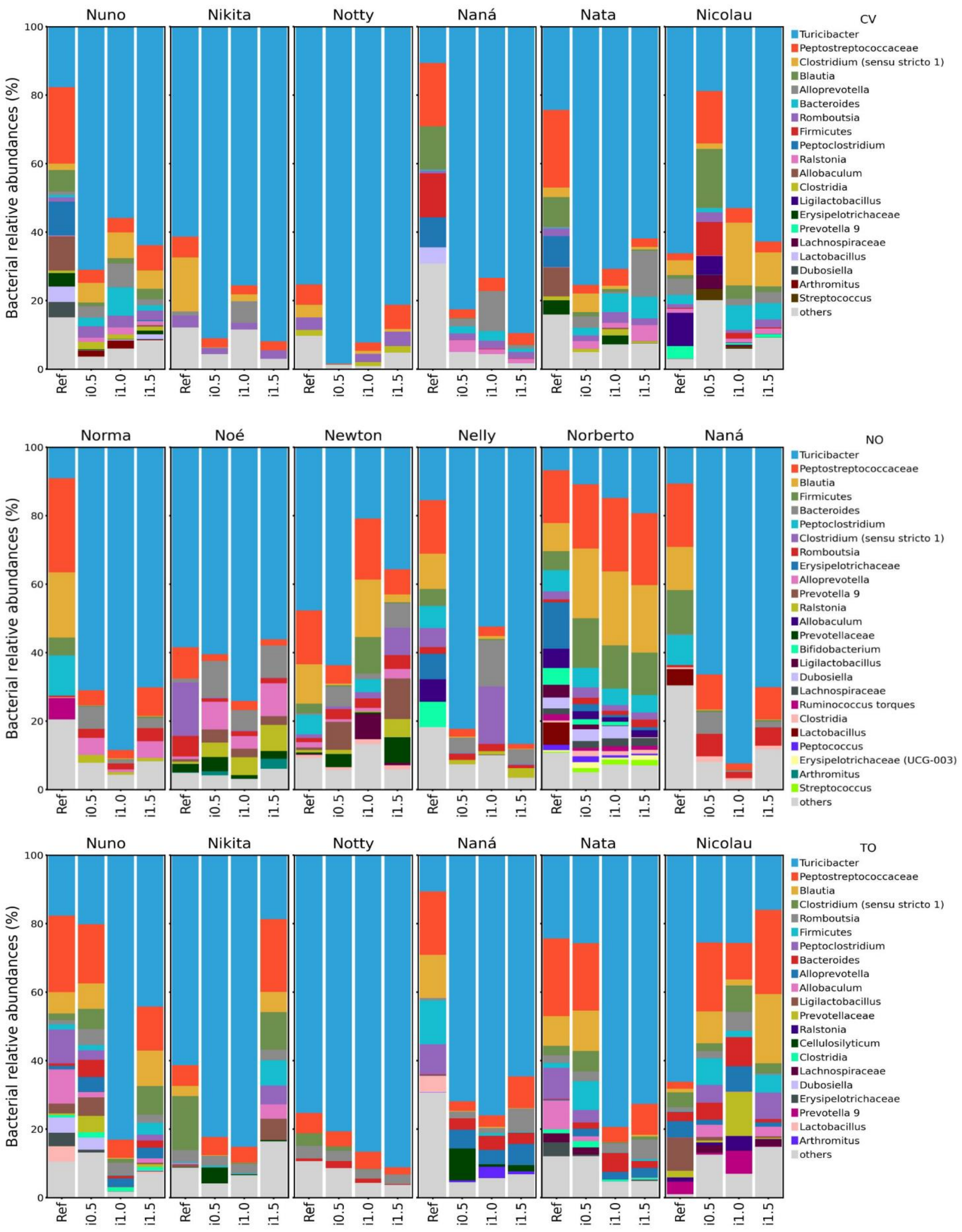
Nuno
Nikita
Notty
Naná
Nata
Nicolau
CV
Bacterial relative abundances (%)
0
20
40
60
80
100
Ref
i0.5
i1.0
i1.5
Turicibacter
Peptostreptococcaceae
Clostridium (sensu stricto 1)
Blautia
Alloprevotella
Bacteroides
Romboutsia
Firmicutes
Peptoclostridium
Ralstonia
Allobaculum
Clostridia
Ligilactobacillus
Erysipelotrichaceae
Prevotella 9
Lachnospiraceae
Lactobacillus
Dubosiella
Arthromitus
Streptococcus
others
Norma
Noé
Newton
Nelly
Norberto
Naná
NO
Turicibacter
Peptostreptococcaceae
Blautia
Firmicutes
Bacteroides
Peptoclostridium
Clostridium (sensu stricto 1)
Romboutsia
Erysipelotrichaceae
Alloprevotella
Prevotella 9
Ralstonia
Allobaculum
Prevotellaceae
Bifidobacterium
Ligilactobacillus
Dubosiella
Lachnospiraceae
Ruminococcus torques
Clostridia
Lactobacillus
Peptococcus
Erysipelotrichaceae (UCG-003)
Arthromitus
Streptococcus
others
Nuno
Nikita
Notty
Naná
Nata
Nicolau
TO
Turicibacter
Peptostreptococcaceae
Blautia
Clostridium (sensu stricto 1)
Romboutsia
Firmicutes
Peptoclostridium
Bacteroides
Alloprevotella
Allobaculum
Ligilactobacillus
Prevotellaceae
Ralstonia
Cellulosilyticum
Clostridia
Lachnospiraceae
Dubosiella
Erysipelotrichaceae
Prevotella 9
Lactobacillus
Arthromitus
others
